# *Pectobacterium atrosepticum* Biosensor for Monitoring Blackleg and Soft Rot Disease of Potato

**DOI:** 10.3390/bios10060064

**Published:** 2020-06-15

**Authors:** Mahdis Hashemi Tameh, Elisabetta Primiceri, Maria Serena Chiriacò, Palmiro Poltronieri, Masoud Bahar, Giuseppe Maruccio

**Affiliations:** 1 Division of Plant Pathology, Department of Plant Protection, College of Agriculture, Isfahan University of Technology, Isfahan 8415683111, Iran; mbahar@cc.iut.ac.ir; 2 Institute of Nanotechnology, CNR-Nanotec, Via per Monteroni, 73100 Lecce, Italy; mariaserena.chiriaco@nanotec.cnr.it (M.S.C.); giuseppe.maruccio@unisalento.it (G.M.); 3 CNR-ISPA, Istituto di Scienze delle Produzioni Alimentari-Consiglio Nazionale delle Ricerche, 73100 Lecce, Italy; palmiro.poltronieri@ispa.cnr.it; 4 Department of Mathematics and Physics “Ennio De Giorgi”, University of Salento, Omnics Research Group, Via per Monteroni, 73100 Lecce, Italy

**Keywords:** lab-on-chip, biosensors, phytopathogen

## Abstract

*Pectobacterium atrosepticum* (Pba) is a quarantine and threatening phytopathogen known as the causal agent of blackleg and soft rot disease of potatoes in many areas. Its early detection is then important to have healthy potato tubers and reduce economic losses. Today, conventional methods such as enzyme-linked immunosorbent-assay (ELISA) and polymerase chain reaction (PCR) are typically used for Pba detection, but they are expensive and time-consuming. Here we report on the optimization of an alternative approach based on an electrochemical impedance immunosensor combining a microfluidic module and a microelectrodes array, and having advantages in terms of low cost, ease of use and portability. For validation and for assessing its performance, the lab-on-chip platform has been compared with two standard methods (ELISA and PCR).

## 1. Introduction

### 1.1. Emerging Needs in Phytodiagnostics

The agrifood field recently exhibited a strong need for new tools to fight pathogen infections by bacteria, fungi and/or viruses, which every year cause worldwide 20% crop losses and 10% post-harvest losses. Improvements are important in order to mitigate the problem and increase food production, taking also into account the present trend in population increase. To reduce crop losses, two actions are possible. The first one consists in the massive use of chemical compounds that has the side effect to increase the pathogen resistance. The second strategy, in line with present initiatives and laws to reduce pesticides use, focuses on improvement of diagnostic capability through the exploitation of new detection systems which permit identifying specific plant disease and applying treatment only when required [1].

*Pectobacterium atrosepticum* (*P. atrosepticum*, Pba) is a plant pathogen belonging to the *Enterobacteriaceae* family, which is predominantly found in temperate regions and leads to blackleg and soft rot diseases of potato. Around the world, *P. atrosepticum* is mainly limited to potato, unlike other blackleg pathogens such as *P. carotovorum* subsp. *carotovorum*, *P. carotovorum* subsp. *brasiliense*, *P. parmentieri*, and *Dickeya* spp. [2,3]. By affecting potato yield, tuber quality as well as food biosecurity, *P. atrosepticum* is one of the most dangerous potato pathogens [2,4] and can lead to significant economic losses ($20–100 million worldwide every year) [2,5,6].

**Figure 1:**
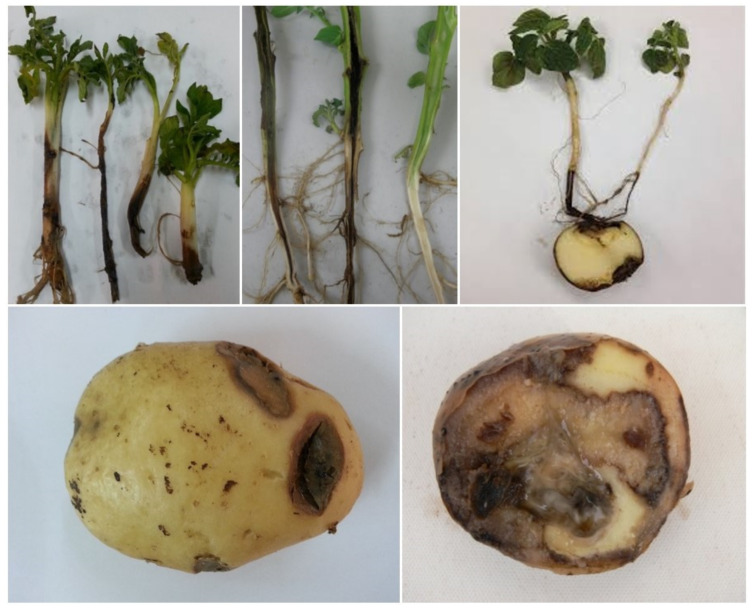
Damage induced by *P. atrosepticum* on seed tuber and stem of potatoes.

The bacterium may be concentrated in potato stems and tubers and live there for a significant time in latent form without showing symptoms, but *P. atrosepticum* can produce huge amounts of extracellular pectinolytic enzymes which leads to specific decay symptoms. This can result in the destruction of the plant cell wall and the middle lamella of leaves, stems, roots, and tubers (Figure 1). As there are not sufficiently effective protocols for mass treatment, one of the best agro-technical approaches is to reject the infected plants. For all these reasons, timely detecting *P. atrosepticum* is crucial to effectively protect plants and produce potato seed tubers [7,8].

Currently, several techniques have been applied to diagnose Pba, including biochemical, immunochemical and molecular approaches [7,9,10]. Laboratory-based diagnostic methods such as culturing methods, enzyme-linked immunosorbent-assay (ELISA), and polymerase chain reaction (PCR) are the gold standard in diagnostics, but require long times, and transfer from the crop to equipped and specialized laboratories with trained personnel. In order to monitor Pba in the field, it would be desirable to have access to faster, simpler and low-cost detection methods.

### 1.2. New Technological Solutions in Phytodiagnostics and POC Testing

Point-of-care (POC) and on-field testing has revolutionized diagnostic methods in several fields included plant testing, offering the possibility to have user friendly tools for quick and on site phytophatogens detection. In this respect, lab-on-a-chip (LOC) devices comply with all these requirements and are gaining popularity in many fields of application, including clinical diagnostics [11,12,13] and agro-food monitoring, representing a valuable alternative also for plant pathogen monitoring [14,15,16,17]. Indeed, recently, this method has been tested to detect quarantine pests such as *Citrus tristeza virus* (*CTV*) [18] and plant pathogens such as the *Xylella fastidiosa* subsp. *pauca* strain, which is related to the olive quick decline syndrome [15]. Furthermore, by integrating sensors with microfluidics tools, LOC makes detection and isolation of bacterial cells possible by performing analytical and pre-analytical procedures on one platform, allowing on-site investigation about possible contamination of plants directly in the field or in customs checkbox to control imported goods.

Among potential technologies to implement sensitive biosensors for pathogens detection, electrochemical impedance spectroscopy (EIS) is particularly attractive because of its sensitivity, ease of miniaturization and low cost [13,15]. Indeed, electrochemical methods can allow bacterial identification and quantification by measuring changes in electrical parameters correlated to the release of ionic metabolites or the capture of bacterial cells by specific antibodies on the electrode surface.

In this study, we report the validation of an impedance LOC for the specific detection of *P. atrosepticum* and the performance of the designed devices is compared with standardized ones (including molecular and immunoassay tests). The platform consists of an array of interdigitated electrodes functionalized with specific antibodies against surface antigens of *P. atrosepticum* and aligned with microchannels and microchambers for fluids handling. The fabrication and structure of such devices is described in detail in the Materials and Methods section together with the protocols used for ELISA and PCR analysis. Then, in the Results and Discussion section, we show that LOC methods can be significantly helpful in monitoring and detecting how *P. atrosepticum* spreads, by providing a convenient inexpensive tool having better performance compared to standard ELISA (enzyme-linked immunosorbent assay) tests. In the conclusions, a comparison with the standard methods and other recent point of care devices is provided in order to demonstrate that the proposed approach is competitive for phytodiagnostics.

## 2. Materials and Methods

### 2.1. Bacterial Samples

Biological source (*P. atrosepticum* SCRI 1039) was obtained from the collection of the James Hutton Institute (Scotland, United Kingdom) and the dilutions in the test ranged from 10^9^ to 10^2^ CFU (colony-forming unit)/mL. Two other bacteria sources belonging to other Pectobacreium and Dickeya species (*Pectobacterium parmentieri* strain Wpp161, *Dickeya solani* strain 2222) were also obtained from the collection of the James Hutton Institute (Scotland, United Kingdom) and used as a negative control for diagnostic specificity. The sets of samples used for ELISA and PCR (Polymerase chain reaction) were the same.

### 2.2. ELISA and PCR Analysis

To assess the performance of the developed LOC, samples were evaluated using ELISA and PCR. Indirect-ELISA tests were carried out using the recommended antiserum (Agdia Inc., Elkhart, IN, USA) while dilutions ranged from 10^9^ to 10^2^ CFU/mL. ELISA cross-reaction was not observed against the plant pathogens: *Agrobacterium tumefaciens; Erwinia amylovora, Pseudomonas marginalis, Ralstonia solanacearum; Xanthomonas campestris, Klebsiella oxycota, Escherichia coli, Rhodococcus rhodochrous, Curtobacterium flaccumfaciens, Pectobacterium parmentieri, P. carotovorum* sub sp *carotovourum*, *P. carotovorum* sub sp *brasiliense*, *Dickeya. Solani*, *D. dadantii*, and *D. zeae*. The absorbance values at 405 nm (OD405) were recorded 4 h after adding the substrate, using an ASYS Hitech GmbH Expert 96 microplate reader. Numbers were normalized as R values (OD-sample/OD-negative control). R = 2.0 was used as a threshold to distinguish between positive and negative responses [18].

Genomic DNA was obtained from bacterial cultures by a standard protocol [19]. Primers ECA1f/ECA2r and Y45/Y46 were used in PCR reactions [20,21]. PCR amplifications were conducted as described using the following thermal regime: 94 °C/10 min, 34 × (94 °C/30 s, 62 °C/45 s, 72 °C/45 s) and 72 °C/10 min. The given primers are specific to the chromosomal DNA of *P. atrosepticum* and the amplification products show 690 bp and 439 bp, respectively. Agarose gel electrophoresis (1.5% agarose gel with ethidium bromide) was used to resolve amplicons.

### 2.3. Lab-On-Chip Platform

**Figure 2:**
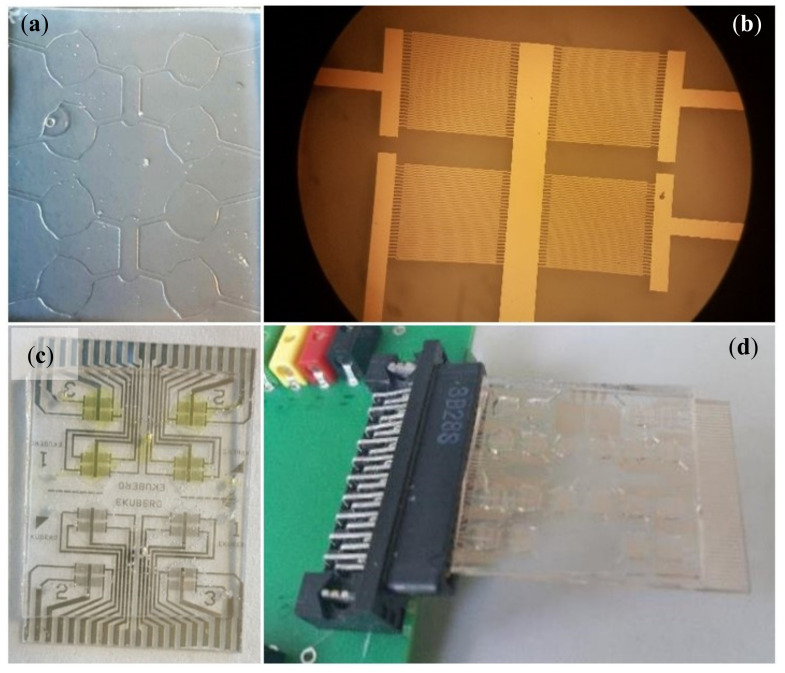
(**a**) Microfluidic modules for fluid handling consisting of four chambers per sides connected by a network of microchannels; (**b**) detail of the four interdigited electrodes available in each sensing area; (**c**,**d**) image of the lab-on-chip and its connection to the interface printed circuit board.

The lab-on-chip optimized to detect *P. atrosepticum* (Figure 2) is similar in structure to the prototypes reported before for other applications [11,15,22,23] and consists of an array of gold interdigitated electrodes for EIS measurements combined to a microfluidic module which allows fluids handling and the confinement of the solutions (reagents for functionalization and sample to analyze). The microfluidic modules with microchannels and microchambers (with a volume of 20 μL) were obtained by PDMS replica molding, from an SU8 hard master. The layout includes a common central inlet and four peripheral outlets per side (half chip) that can be utilized to independently functionalize each chamber or to inject the sample to be tested in quadruplicate improving reproducibility and allowing to save reagents and samples (Figure 2a). The electrodes have a line-space period of 10 μm and cover a 2 mm × 1.5 mm area. They were fabricated by optical lithography (using a Karl Suss MJB3 mask aligner and AZ5214e resist) and thermal evaporation of Cr/Au (3 nm/15 nm) (Figure 2b). Here, highly specific antibodies (the same used for ELISA tests from Agdia Inc., Elkhart, IN, USA) were employed to functionalize the microelectrodes in order to make them capable to bind specifically to *P. atrosepticum* cells. Particularly, the functionalization includes the following layers and steps:

(1) a mixed self-assembled monolayer (SAM) of beta-mercaptoethanol and mercaptoundecanoic acid obtained by overnight incubation in ethanol solution;
(2) the activation of the COOH groups of 11-mercaptoundecanoic acid (MUA) by incubation for 30 min in a solution of N-hydroxysuccinimide and N-ethyl-N-3-dimethylaminopropyl carbodiimide hydrochloride at a ratio of 1:4 in milliQ water;
(3) a protein A monolayer assembled by covalent binding to the mixed SAM by a 2 h incubation in a (50 μg/mL) phosphate buffered saline (PBS) solution;
(4) the disactivation of ester reactive groups by incubation in ethanolamine (1 M);
(5) the passivation of the unbound surface by incubation in bovine serum albumin (BSA) (1 mg/mL);
(6) the oriented immobilization of the antibodies specific for *P. atrosepticum* (5 ng/mL) by binding to protein A through their Fc region (fragment crystallizable region).

In this way, protein A achieves a covalent binding to SAM of mercapto undecanoic acid. Then Protein A naturally binds the Fc region of antibodies allowing their oriented immobilization. Ethanolamine was used to block reactive groups while BSA was used to passivate and saturate the unbound surface. As the final step, each sensing area was functionalized using antibodies.

All reagents were purchased from Sigma-Aldrich Inc. (St. Louis, Missouri, USA), except the antibodies.

The calibration tests were carried out by known dilutions of bacteria suspensions in PBS to evaluate the sensitivity of the assay. For this purpose, bacteria cell dilutions ranging from 10^9^ CFU/mL to 10^4^ CFU/mL were delivered through the microfluidic channels and left in static conditions into the microchambers for 1 h to allow the biorecognition between the suspended cells of *P. atrosepticum* and antibodies immobilized on the surface. Afterward, 1 mL of washing PBS solution was delivered and chambers were filled with a solution of hexacyanoferrate (II/III) K_3_[Fe(CN)_6_]/K_4_[Fe(CN)_6_] (1:1) at a concentration of 10 mM to measure the electrochemical impedance with an Autolab PGSTAT30 under an applied sinusoidal 15 mV AC voltage at a frequency ranging from 10^5^ Hz to 0.1 Hz. Impedance spectroscopy raw data were exported as ASCII files and OriginPro 2018 (OriginLab Corporation, Northampton, MA, USA) was used to process them.

## 3. Results and Discussion

### 3.1. ELISA and PCR Assays

In ELISA assays (Table 1), cross-reaction against other plant pathogens and other species of *Pectobacterium* and *Dickeya* was not observed. Positive readings up to a dilution of 10^5^ CFU/mL were obtained. The R value of positive control was 33, while the negative control yielded an absorbance value of less than 0.06.

**Figure 3:**
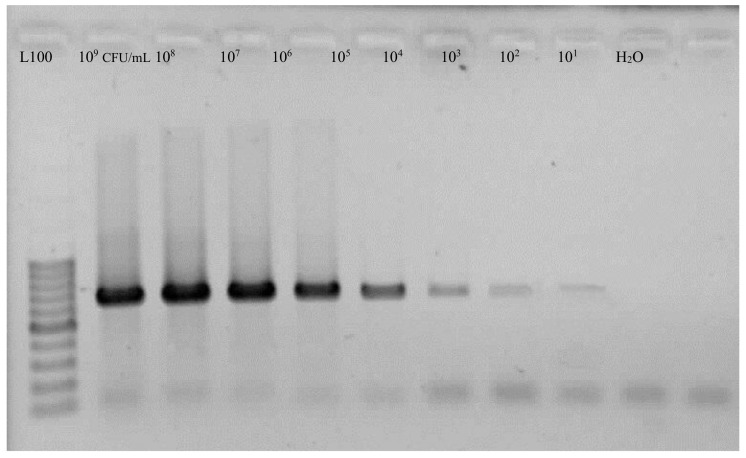
Agarose gel electrophoresis of polymerase chain reaction (PCR) sensitivity on bacterial isolates by Y45/Y46 primers.

In PCR assays, to determine the specificity of the primer sets, tests were performed using Eca1/Eca2 and Y45/Y46 primers with DNA from all samples. Two 690 bp and 439 bp DNA fragments were respectively produced for *P. atrosepticum* isolates, while no amplification was observed with isolates of other *Pectobacterium* spp. and *Dickeya* spp. Different concentrations of Pba isolates were used and the estimation for PCR sensitivity was 10^2^ CFU/mL (Figure 3).

### 3.2. LOC Detection

**Figure 4:**
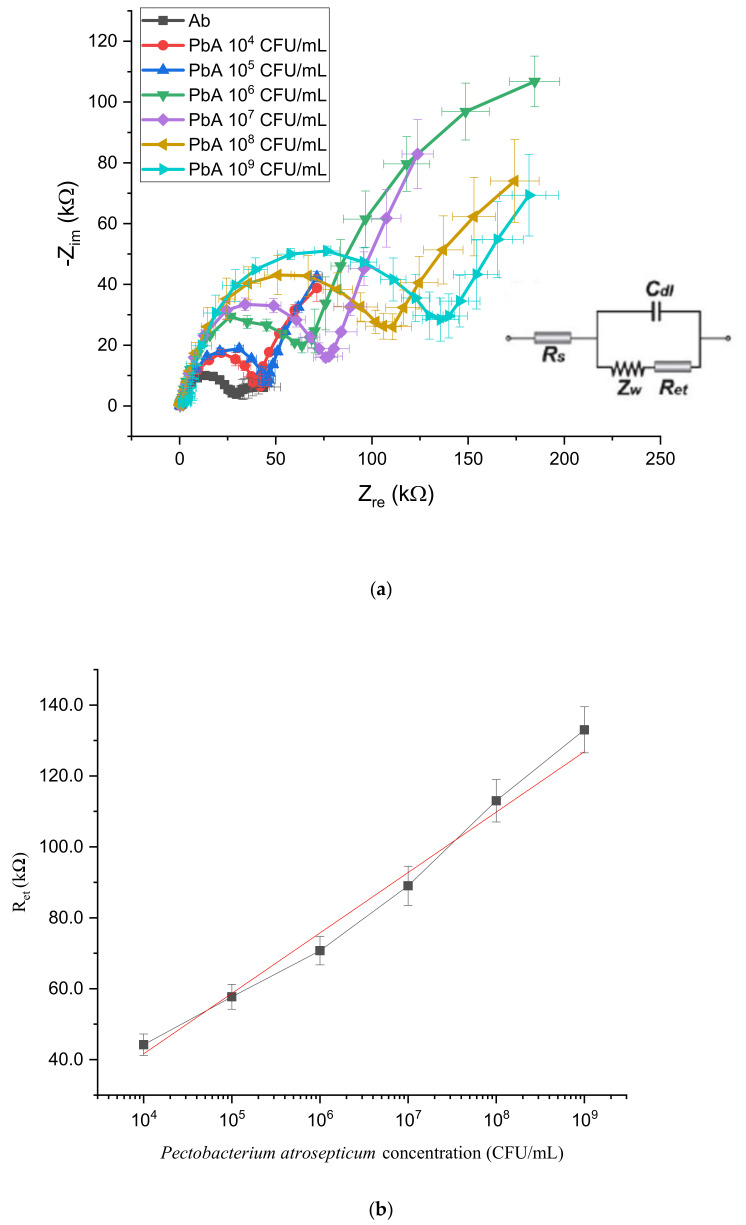
(**a**) Nyquist spectra obtained in lab-on-a-chip (LOC) device calibration. Curves from cyan to red correspond to different concentrations of *Pectobacterium atrosepticum* in spiked solutions; each curve is the average of three different measurements. (**b**) Impedance values as a function of *Pectobacterium atrosepticum* concentration: each point is the average of three different measurements.

To evaluate the sensitivity of the designed EIS lab-on-chip, impedance spectroscopy measurements were carried out in a frequency ranging from 10^5^ to 0.1 Hz and plotted as Nyquist curves where the *x*-axis is the real component of impedance Z_re_ while the *y*-axis is the complex component −Z_im_. The system can be modeled by an equivalent (Randles) circuit (inset of Figure 4a), describing the interfacial layer at the working electrode as an electron transfer resistance R_et_ in parallel combination with an electrical double layer capacitance C_dl_. A series Warburg impedance Z_w_ accounts for redox species depletion at the interface, while R_s_ is the uncompensated solution resistance. The electron transfer resistance (R_et_) is very sensitive to electrode modifications and roughly corresponds the diameter of the semicircle-shaped curves.

As a first step, the sensing layer consisting of antibodies immobilized on the electrode surface was characterized to identify their impedance contribution. Anti- *P. atrosepticum* antibodies layer resulted in R_et_ ≈ 30 kΩ (dark gray line in Figure 4a). Then, to calibrate the sensors response, serial dilutions of *P. atrosepticum* in PBS (10^9^, 10^8^, 10^7^, 10^6^, 10^5^, 10^4^ CFU/mL) were prepared and tested. After incubation and washing steps, redox solution was added in the chamber and EIS measurements were collected. As shown in Figure 4a, a noticeable rise in impedance values with respect to the antibody baseline can be associated with incubation with a spiked solution on the sensing layer (colored curves) and impedance values are correlated with the bacterial *P. atrosepticum* concentration [C] in suspension (linearly in a R_et_ vs. log [C] plot) whose decrease results in smaller R_et_ values (Figure 4b). The suspension containing 10^4^ CFU/mL was associated with R_et_ ≈ 42 kΩ (red curve), while the most concentrated suspension with 10^9^ CFU/mL resulted in R_et_ ≈ 135 kΩ (light blue curve).

**Figure 5:**
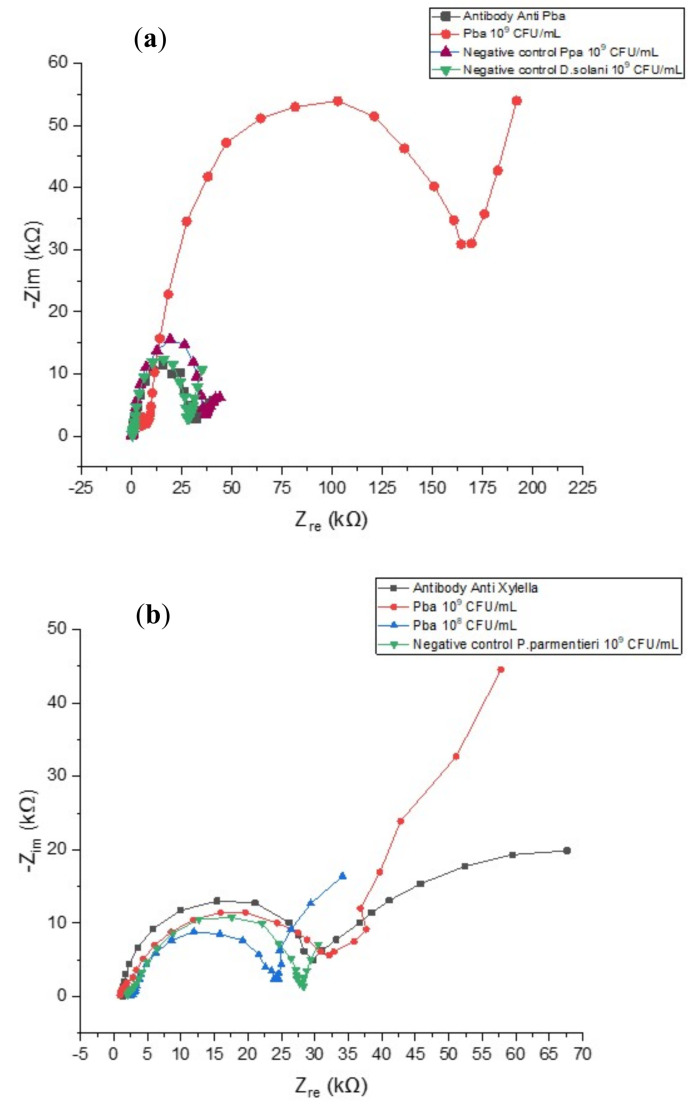
(**a**) Nyquist spectra obtained during negative testing with *P. parmentieri* and *Dickeya solani* at 10^9^ CFU/mL and positive control (*Pectobacterium atrosepticum* 10^9^ CFU/mL). (**b**) Nyquist spectra obtained by testing of *P. atrosepticum*, *P. parmentieri* and *D. solani* on a chip functionalized with antibodies anti-*Xyllella. fastidiosa*.

As a negative control, we incubated *P. parmentieri* or *Dickeya solani* suspensions containing 10^9^ CFU/mL. As shown in Figure 5a, impedance curves obtained in these cases (purple and green curves respectively) resulted in R_et_ values close to the antibody baseline (≈30 kΩ) while the same concentration of Pba gave R_et_ ≈ 175 kΩ, demonstrating the high specificity of the optimized sensing system.

To further evaluate specificity of our LOC and a possible aspecific interaction between the bacterial cells and the electrode’s surface, we also immobilized a different antibody (Ab anti-*Xyllella. Fastidiosa*) not able to specifically interact with Pba. In this case, we obtained R_et_ ≈ 30 kΩ by adding different concentrations of *P. atrosepticum*, *P. parmentieri* and *D. solani*, showing again the high specificity of the LOC assay (Figure 5b).

## 4. Conclusions

Conventional diagnostic techniques usually need a long time for processing and a great deal of effort. Detection methods based on immunological approaches such as ELISA can be automated and therefore become effective in terms of time and effort, but there are some limitations which include low sensitivity and false-positive results. In the present work, a reliable, inexpensive and label-free LOC assay has been set to detect *P. atrosepticum* down to 10^4^ CFU/mL. As reported by Czajkowski et al. [7], the usual level of lead accumulation in infected tubers is 10^5^ and the density of bacteria in infected tissues is often more than 10^6^, which is higher than the limit of detection of our device. Therefore, according to these results, the LOC can be used to detect Pba in infected tubers.

In Table 2, LOC sensitivity is compared to ELISA and PCR, demonstrating competitiveness of the developed assay as it provides a better sensitivity than the ELISA method. Indeed, ELISA tests are not able to detect pathogens at dilutions below 10^5^ CFU/mL, while the developed LOC platform could identify bacteria cells down to 10^4^ CFU/mL dilutions. On the other hand, PCR methods showed better performances compared to LOC devices with positive cases identified even at 10^2^ CFU/mL, but this nucleic acid-based method is expensive and advanced equipment and experienced technicians are needed to perform it. The introduced device can provide a good compromise considering the need for sensitivity and assay accessibility. As mentioned earlier, the developed LOC assay also has a good specificity since it can detect 10^4^ CFU/mL of Pba (below the typical 10^5^ CFU/mL value in infected tubers) and differentiate it from other *Pectobacterium* and *Dickeya* species. Another advantage of the proposed LOC devices is associated with cost. Based on our estimations, it is almost 1.8 to 6 times less expensive than ELISA and PCR. The major factors contributing to the costs of LOC devices (around 5 €/device) are mainly the glass substrate and the metallization, since the volumes of employed reagents are very low with minimal use of antibodies.

Another useful comparison can be done with recent devices proposed for plant disease diagnosis. As shown in Table 3, the performance of our sensing platform is competitive with respect to other recent technologies in terms of sensitivity and the limit of detection. The two techniques based on lateral flow immune assays offer the great advantage to be very cheap and simple, two key aspects for on field analysis, but on the other hand they are not quantitative [24,25]. Papadakis and coworkers proposed an approach based on quartz crystal microbalance to detect DNA amplificons and they showed a LOD of 10^3^ CFU/mL that is lower than our devices but their approach requires additional steps for DNA extraction from plant tissues and DNA amplification [26], which results in an increase in the cost and time necessary for the analysis. In the last work, Yazgan and coworkers optimized two sensing approaches based on cyclic voltammetry and surface plasmon resonance by using carbohydrate ligand as the bioreceptor. They modified with different moieties the ligands and obtained a very sensitive tool with improved LOD down to 1 CFU/mL with the SPR approach, but the limit of this smart solution is related with the possibility of cross reaction with other bacterial strains and the difficulties to translate an SPR approach into a point of care device [27].

On the contrary, our sensing platform is particularly suitable for POC applications thanks to its limited dimension (a few square centimeters). In addition, the number of sensors in the array can be increased by further miniaturization, permitting more analysis on the same chip without affecting its cost and enabling the simultaneous detection of several pathogens ranging from viruses to bacteria and fungi.

As next steps towards a fully integrated platform for POC phytodiagnostics, a read-out board for electronic connections with a portable potentiostat can be employed enabling a new and smart approach for on field plant diagnostics, including the use of a smartphone for data acquisition and sharing. In fact the current trend in automation in agriculture makes it more and more difficult to perform a manual inspection of plants in the crops for disease monitoring, so the use of smartphones and wireless technology together with the new developing internet of things (IoT) [28] open new frontiers in agriculture [29], offering the possibility to collect and analyze data about the diffusion of a disease in order to map the spreading of the pathogens and, for example, prevent the infection of healthy surrounding areas.

## Figures and Tables

**Table 1:** Results of enzyme-linked immunosorbent-assay (ELISA) tests (− = negative, + = positive) on samples ranging from 10^9^ to 10^2^ CFU mL^−1^ (where CFU = colony-forming unit), R = OD-sample/OD-negative control.

CFU/mL	ELISA		
	OD	R	Result
10^9^	2	33	+
10^8^	1.8	30	+
10^7^	1.12	18.67	+
10^6^	0.21	3.5	+
10^5^	0.16	2.6	+
10^4^	0.088	1.47	-
10^3^	0.063	1.05	-
10^2^	0.034	0.56	-
10^1^	0.015	0.25	-
Control	0.06	1	-

**Table 2:** Performance comparison: LOC is more effective in detecting *P. atrosepticum* compared to the ELISA method, while PCR results in higher sensitivity, but at larger costs.

Technique	Limit of Detection
ELISA	10^5^ CFU/mL
PCR	10^2^ CFU/mL
LOC	10^4^ CFU/mL

**Table 3:** Performance comparison with other devices for plant disease diagnosis.

Detection Technique	Bioreceptor	Plant	Bacteria	LOD	Ref.
Lateral Flow Immunoassay	Ab	Potato	*Dickeya dianthicola* and *Dickeya solani*	4 × 10^5^ CFU/mL	Safenkova et al. [25]
Quartz Crystal Microbance	DNA	tomato	*Ralstonia solanacearum*, *Pseudomonas syringae* pv. *tomato*, *Xanthomonas campestris* pv. *vesicatoria*	10^3^–10^4^ CFU/mL	Papadakis et al. [26]
Cyclic Voltammetry	Modified carbohydrate ligand	Spinach	*E. coli*	6.25 × 10^2^ CFU/mL	Yazgan et al. [27]
Surface Plasmon resonance	Modified carbohydrate ligand	Spinach	*E. coli*	1–10^4^ CFU/mL	Yazgan et al. [27]
Lateral Flow immunoassay in multiarray on a test strip (MATS) format	Ab	Potato	*Clavibacter michiganensis*	10^4^ CFU/mL	Safenkova et al. [24]
